# Dental Pathology and Surgery

**Published:** 1875-03

**Authors:** 


### Book Notices.

DENTAL PATHOLOGY AND SURGERY. By S. JAMES H. SALTER, M. D., F. R. S. Published by Wm. Wood & Co. For sale by Robert Clarke & Co., 65 West Fourth street, Cincinnati, O.

This volume is made up from the papers and essays of the above named author, and contains the results of his researches and labors for the past twenty-three years. It places in a form that is available much valuable information, both instructive and interesting, many important points of practice which could not be obtained without a troublesome and tedious reference to a general medical and surgical library; and there are a large number of dentists who are not in reach of such. Mr. Salter has been a constant investigator and writer during his entire professional career. His articles have been distributed throughout medical and dental literature, so that in collecting and arranging them in their present shape he has done a service that will be productive of much good. The work is purely what its title represents it to be, and is not in any way mixed up with the mechanics of dentistry. It is a valuable work for consultation and reference, and should be in the library of every dentist.

